# Editorial: Insects at the Center of Interactions With Other Organisms

**DOI:** 10.3389/fphys.2020.00616

**Published:** 2020-06-23

**Authors:** Anne-Nathalie Volkoff, Michel Cusson, Patrizia Falabella

**Affiliations:** ^1^INRAE Institut National de Recherche pour l'Agriculture, l'alimentation et l'Environnement, Montpellier, France; ^2^Laurentian Forestry Centre, Natural Resources Canada, Quebec City, QC, Canada; ^3^Department of Sciences, University of Basilicata, Potenza, Italy

**Keywords:** biomimicry, insects, behavior, ecology, biotechnology, environment

Insects represent the largest and most diverse group of living organisms and are involved in a wide spectrum of interactions with other organisms. Many such interactions have been the focus of scientific investigations, including some reported here in a collection of *Frontiers in Physiology* papers published under the Research Topic “Insects at the center of interactions with other organisms.” Below is an overview of the subject matters and key findings of each article included in this collection. As you will come to appreciate, these studies are very diverse, both in terms of the subject organisms and research tools used to shed light on the interactions under investigation, but all of them report on cases in which insects are at the center of interactions with other organisms.

## Insects as Model Organisms for Biological and Biomedical Studies

Insects, with over 1 million described species, have a worldwide distribution and display an amazing ability to adapt to all types of environmental conditions so long as in presence of organic substances as a food source. In addition, their breeding is easy and inexpensive and several of their biochemical pathways at the cellular level are very similar to those of vertebrates, allowing us to extrapolate some findings about their biology and physiology to vertebrates (a famous case being research on drosophila development and immunity (Anderson and Nusslein-Volhard, 1984; Lemaitre et al., 1996), which led to the discovery of Toll-like receptors in humans). For these reasons, many insects have been used as model organisms in biological, medical and environmental research. In recent years, species in the order Coleoptera have increasingly been used as model organisms in biomedical and environmental studies. In this Research Topic, Adamsky et al. review current knowledge regarding the use of beetles as model organisms and the practical applications resulting from it to various fields of the life sciences.

## Host-Parasitoid Interactions

Parasitoid wasps represent one of the most abundant and diversified groups of insects on Earth, and interactions between parasitoids and their invertebrate hosts have been extensively studied. Parasitoids have long fascinated researchers as biological models to explore host-parasite co-evolution, insect development and immunity, insect behavior, or have been studied because they are natural enemies of insect pests and thus widely used as biological control agents.

Parasitoids exhibit different life habits and widely diverse interactions with their hosts. Complex physiological interactions are found between parasitoids that develop within a living insect (so called koinobiont endoparasitoids). Because of this particular life cycle, this group of parasitoids have developed fine-tuned strategies allowing them to deal with their host's immunity, development or metabolism in order to allow proper development of their progeny. Strategies include injection into the host, by the female wasp, of venom proteins or viral particles produced in the genital track. Acquisition of knowledge on the molecular mechanisms underlying these complex interactions has largely benefitted, in the recent years, from the emergence of high-throughput technologies combined with more classical biochemical and cellular biology techniques. For instance, the whole arsenal of proteins produced in the venom gland of drosophila parasitoids in the genus *Leptopilina* has been elucidated thanks to a combined transcriptomic and proteomic approach. A review of the advances achieved in this field is presented in a paper by Kim-Jo et al. The review also highlights how the field of parasitoid “venomics” contributed to advance knowledge of insect cellular immunity. Similarly, knowledge on the mutualistic viruses produced in the ovaries of braconid and ichneumonid wasps has considerably advanced in recent years. The viral particles are injected into a caterpillar host upon oviposition by the female wasp. The packaged viral genome includes hundreds of genes that are expressed in the parasitized insect where their expression leads to alterations of the physiology of the parasitoid's host. Two papers present novel data on how the parasitized insect is affected in these biological systems and thus contribute to an understanding of how koinobiont parasitoids regulate the physiology of their host to ensure the development of their progeny. A first study by Merlin and Consoli analyses the transcriptome of caterpillars parasitized by the virus-carrying braconid wasp *Cotesia flavipes*, allowing them to establish an extensive list of lepidopteran genes potentially involved in host immune response, metabolism and development, and showing that transcription is strongly affected by parasitism. Another study by Salvia, Nardiello et al. investigates the manipulation of host development by the braconid wasp *Toxoneuron nigriceps* using different approaches; the authors, here, nicely demonstrate the involvement of viral proteins in blocking ecdysteroidogenesis in host prothoracic glands, through alterations of the PI3K/Akt/TOR pathway at the transcriptional level. Finally, beyond maternal factors, host suitability may be promoted by factors released by the developing, immature wasp. Some parasitoid species, upon egg hatch, release unusual giant serosal cells called teratocytes, responsible, among other functions, for extra-oral digestion of host tissues. In this issue, using biochemical and cellular biology approaches, Salvia, Grimaldi et al. report on the unconventional mechanism by which teratocytes from the braconid wasp *Aphidius ervi* release enolase and fatty acid binding proteins, and reveal that these proteins are probably released via an exosome secretion pathway.

## Insect-Nematode Interactions

With a parasitic life history similar to that of parasitoid wasps, parasitic nematodes also develop within living insects. To complete their life cycle, nematodes rely on symbiotic bacteria responsible for the death of the insect, the nematode then feeding on the insect cadaver. Intricate interactions also exist between the insect and the complex formed by the nematode and its mutualistic bacteria. In this issue, Ozakman and Eleftherianos provide insights into the signaling pathways that are activated in insects when they are infected with entomopathogenic nematodes; they show, using mutant flies, that lipid metabolism is affected in nematode-infected insects via the regulation of the activin and BMP branches of TGF-ß signaling.

## Insects and Their Interactions With Endosymbionts

Bacterial symbionts of insects are very common and, in many instances, can have profound effects on the physiology of their insect hosts. The reality of their prevalence is highlighted by the recent characterization of specific microbial communities in many insects, thanks to new genomic sequencing technologies. Plant sap-sucking insects form a well-studied group in terms of their association with primary symbionts, which supply them with essential nutrients; these insects are also known to harbor diverse secondary symbionts. In addition to their role in insect nutritional ecology, bacterial symbionts can mediate several other ecologically relevant traits, including defense toward pathogens and parasites, adaptation to environment, influences on insect-plant interactions, and impact on population dynamics (for an overview see Su et al., 2013). In the Research Topic, several contributing papers explore the role of endosymbionts in a variety of plant sap-sucking insect life traits, using different approaches. A study by Li et al. examines the effects of the secondary bacterial symbiont *Cardinium* on the ecological adaptation of whiteflies. To this end, they analyze differentially expressed miRNAs between infected and uninfected insects; their results lead to the conclusion that symbionts can improve the fitness of their host by increasing its tolerance to environmental stresses via miRNA regulation of gene expression. In another paper, Skaljac et al. show the importance of symbionts in insect-plant interactions. Through an analysis of tissue localization of the secondary bacterial symbiont *Serratia symbiotica* in pea aphid, the authors reveal the presence of the symbiont in several tissues from the aphid's digestive tract, including the salivary glands and the stylet. Consequently, the bacteria were also detected in plant tissues, where they could, by secreting proteases, facilitate digestion of plant proteins and alter plant defense. A third paper addresses the role of symbiotic bacteria in host defense against pathogens. Gonella et al. analyzed the expression of immune genes in the sugar-feeding leafhopper *Euscelidius variegatus*, following exposure to the secondary bacterial symbiont *Asaia*. They conclude that a component of the Ras/Raf pathway is activated by *Asaia* and could potentially mediate the role the symbiont plays in limiting phytoplasma acquisition by the insect.

## Insects as Vectors of Plant Pathogens

Because vector-borne plant diseases can have severe economic and environmental impacts, the factors responsible for, or limiting, their transmission have received much research attention. Insects can vector many plant diseases, including viruses, bacteria and fungi. Consequently, the molecular mechanisms underlying insect-phytopathogen interactions have been intensively studied. Two papers in this Research Topic address the factors that may affect transmission of the tomato yellow leaf curl virus (TYLCV) by whiteflies. These factors include insect vector proteins, as shown by Kanakala et al.. Using bioassays and RNAi technology to knock-down two whitefly gene products previously shown to interact with the TYLCV in the insect midgut, the authors confirmed the role of cyclophilin B and heat shock protein 70 in the whitefly's ability to transmit the virus. Chen G. et al. examined another factor that can affect virus transmission by insects. The authors compared transmission by whiteflies and western flower thrips of two persistently transmitted viruses that can co-infect the two insect species. Their results indicate that presence of TSWV reduces the ability of whiteflies to transmit the TYLCV, and inversely, that the presence of TYLCV in thrips reduces the transmission of TSWV, thus showing a negative effect of co-infections on virus transmission in both biological systems.

Vector-borne plant viruses may also have direct or indirect effects on the insect vectors. Ding et al. explored the molecular mechanisms underlying the response of whiteflies to the plant pathogens they vector (TYLCV, ToCV, TYLCV&ToCV co-infection), using a transcriptomic approach. They identified a list of insect genes that are differentially regulated in infected and non-infected insects. Studying this type of system from the angle of insect nutrition, Guo et al. assessed the effects of TYLCV infection on nutrient availability for different whitefly biotypes. The authors show that TYLCV infection of tomato plants significantly affected the amount of free amino acids in the phloem sap of the tomato plants and of free amino acids in whitefly honeydew. The authors also show that the better performance of the Q biotype is related to its superior ability to obtain free amino acids from the virus-infected plants.

Insects can also be indirect vectors of plant diseases and can thus contribute to emergence of new pandemics. A paper by Santini and Battisti, based on an analysis of three major pandemics affecting forest and urban trees in temperate ecosystems, illustrates how such pandemics can be initiated by the introduction of a non-native pathogen that exploits well-developed interactions between native non-aggressive organisms and insects associated with trees.

## Insect and Plant Trophic Interactions

Herbivorous insects often have intricate relationships with their host plants. Insects rely on a series of chemical cues to locate their host plant, especially plant volatiles such as terpenoids and other compounds emitted from leaves; their response to host plant odors varies with the physiological status of both the plant and the insect. Indeed, plants perceive damage-associated and insect-associated molecular patterns via receptors that activate diverse signaling networks resulting in local and systemic responses. Several papers in the present collection provide insights into the complex interactions between insects and plants and how they affect tritrophic interactions in agro-ecological systems. Cambier et al. explored the molecular mechanisms underlying cynipid gall induction in woody plants. By analyzing the transcriptome of ovaries and venom glands of two cynipid wasps, the authors identified candidate proteins involved in gall induction, including cellulases and potential plant defense suppressors. Bari et al. analyzed the communication strategies adopted by adults of the Mediterranean flat-headed root-borer. By combining morphological studies, bioassays, chemical and transcriptomic analyses, the authors discovered that females regulate mate recognition and acceptance via pronotal secretions related to volatiles emitted from their host plant. Finally, two papers examined interactions between parasitoids (third trophic level) and volatiles emitted by the plant in response to insect damage, and the factors affecting these interactions. Bertoldi et al. examined the role of host-parasitoid coevolution in the “efficiency” of this interaction. Their results indicate that the egg parasitoid *Trissolcus japonicus* efficiently exploits volatiles produced by tomato plants following egg deposition and infliction of feeding punctures by its coevolved host *Halyomorpha halys*; however, it does not respond to cues associated with a novel host, *Podisus maculiventris*. The study by Salerno et al. contributes to elucidating the source and the nature of the elicitor responsible for the recruitment of the egg parasitoid *Trissolcus basalis* by emitting plants following oviposition by stink bugs. By combining olfactometer observations and protein analysis, they identified a candidate protein specifically produced in response to mating in the dilated portion of the stink bug's spermathecal complex.

## Effects of Biotic and Abiotic Factors on Insect Life Traits

Insects live in a wide variety of environments and are therefore exposed to multiple biotic and abiotic factors that may seriously impact on their life traits. Understanding how these factors affect, positively or negatively, insect life traits can shed light on their population ecology and dynamics and, *in fine*, may lead to the development of novel sustainable pest control strategies. The effects different factors have on insect life traits are addressed in this Research Topic; these factors include the nutritional characteristics of their food sources and the presence of pesticides in the environment. Abedi et al. evaluated different commercial cultivars of pomegranate on life traits of a major pomegranate pest, the carob moth. Through biochemical analyses and bioassays, they show that biochemical characteristics of cultivars significantly affect life history traits and demographical parameters in the pest. Results led to a classification of the pomegranate cultivars as susceptible or resistant, a knowledge that is critical to the development of integrated pest management practices. The effect of diet was also investigated by Chen E. et al. who evaluated its impact on susceptibility of the cabbage looper to the AcMNPV baculovirus. Microscopic observations and transcriptomic analyses clearly showed an effect of diet composition on the thickness of the peritrophic membrane of the caterpillars' midgut, an effect that results from differences in chitin deacetylase and chitinase expression, which in turn has consequences for looper susceptibility to the baculovirus. Pesticides can negatively affect beneficial insects, and in particular pollinators. To assess the global impact of pesticides on pollinators, methods for residue detection are needed. Favaro et al. present a practical and reliable approach to identify the source of pesticide residues from pollen collected by honeybees based on pollen and multi-residual chemical analyses. Thanks to an experimental setup at the landscape scale, the authors were able to identify various sources of contamination during the apple blossom season. In addition to their detrimental effects on beneficial insects, pesticides directed against one organism can have unexpected effects on another organism. For instance, Ge et al. provide evidence that applications of the antibiotic jinggangmycin, normally used to control a rice fungal pathogen, stimulates planthopper reproduction via an upregulation of an UDP-glucuronosyltransferase in the insect, which is shown to be a positive modulator of the insect's reproductive biology. This paper underscores the importance of assessing the side effects of a given control product on the entire community of the targeted pest, under natural conditions.

## Microorganisms for the Defense of Plants From Phytophagous Insects

Plant defenses can also be triggered by microorganisms. Fungi in the genus *Trichoderma* are efficient inducers of systemic resistance in plants (Bisen et al., 2016) and largely used as biocontrol agents against plant pathogens. In addition to their role in promoting plant growth, nutrient uptake, and induction of plant defense responses against different biotic and abiotic stresses (Zehra et al., 2017), *Trichoderma* fungi also protect the plant from herbivorous insects, as shown in two papers of this Research Topic by Coppola, Cascone et al. and Coppola, Diretto et al. In these two studies, each conducted using a different *Trichoderma* species, the authors used transcriptomics, proteomics and bioassay approaches to show that treatment with the biological control agent induces transcriptional changes for a wide array of defense-related genes in tomato plants, but also alters plant metabolic pathways. These alterations result in better protection of the tomato plant against two major tomato insect pests (an aphid and a noctuid moth). Interestingly, the treatments also lead to the release of volatile organic compounds that attract aphid parasitoids.

We hope you enjoy reading the articles in this collection!

## Author Contributions

MC, PF, and A-NV wrote, edited, and finalized this manuscript. All authors contributed to the article and approved the submitted version.

## Conflict of Interest

The authors declare that the research was conducted in the absence of any commercial or financial relationships that could be construed as a potential conflict of interest.
